# Genome-Wide Mining of Chitinase Diversity in the Marine Diatom *Thalassiosira weissflogii* and Functional Characterization of a Novel GH19 Enzyme

**DOI:** 10.3390/md23040144

**Published:** 2025-03-26

**Authors:** Mengzhen Cheng, Shuang Li, Jiahui Wang, Xiaoqi Yang, Delin Duan, Zhanru Shao

**Affiliations:** 1 State Key Laboratory of Breeding Biotechnology and Sustainable Aquaculture, Institute of Oceanology, Chinese Academy of Sciences, Qingdao 266000, China; chengmengzhen@qdio.ac.cn (M.C.);; 2 Laboratory for Marine Biology and Biotechnology, Qingdao Marine Science and Technology Center, Qingdao 266237, China; 3 University of Chinese Academy of Sciences, Beijing 100049, China

**Keywords:** β-chitin, chitinase, gene family, glycoside hydrolase 19, diatom, enzymatic activity

## Abstract

Chitin represents a globally abundant marine polymer with significant ecological and biotechnological value. β-chitin is an important carbon fixation product of diatoms and has a greater range of applications than α- and γ-chitin. However, there has been a paucity of research on the characterization of chitin-related enzymes from β-chitin producers. In this study, we performed a genome-wide identification of 38 putative chitinase genes in *Thalassiosira weissflogii*, a key producer of β-chitin. Through comprehensive analyses of phylogenetic relationships, conserved motifs, structural domains, and subcellular localization predictions, we revealed that *T. weissflogii* possesses evolutionarily distinct GH18 and GH19 chitinase families exhibiting unique motif and domain configurations. Subcellular localization predictions showed that most TwChis were presumed to be located in the chloroplast, with a few being present in the nucleus and extracellular. The enzymatic activity of TwChi2, a GH19 chitinase, showed that TwChi2 was a member of exochitinase (EC 3.2.1.201) with strong thermal stability (40 °C) and broad substrate adaptability of hydrolyzing bipolymer, 1% and 5% colloidal chitin, α-chitin and β-chitin. Altogether, we analyzed the chitinase gene family and characterized a highly active exochitinase from *T. weissflogii*, which can catalyze the degradation of both chitin polymers and chitin oligosaccharides. The relevant results lay a foundation for the internal regulation mechanism of chitin metabolism in diatoms and provide a candidate enzyme for the green industrial preparation of high-value chitin oligosaccharides.

## 1. Introduction

Chitin, a polymer of β-(1,4)-linked *N*-acetylglucosamine (GlcNAc), is the most abundant yet underutilized renewable natural resource in the ocean, widely found in marine mollusks, crustaceans, and diatoms [1,2,3]. Chitin is mainly classified into three distinct crystalline forms, designated as α-, β-, and γ-forms [4]. The isotropic parallel glycan chain segment structure of β-chitin results in weaker intermolecular forces, thus endowing β-chitin with a wide range of ecophysiological roles and potential applications. Despite these advantages, there has been a paucity of research on the characterization of chitin-related enzymes from β-chitin producers [5]. The centric diatom genera *Thalassiosira* and *Cyclotella* are the main producers of β-chitin [6,7,8,9]. It has been reported that *T. weissflogii* has a robust capacity for chitin biosynthesis, which is an exemplary material for investigating chitin-related enzyme resources and chitin derivatives [10,11].

The biosynthesis and degradation of chitin play a pivotal role in the global biogeochemical cycling of carbon and nitrogen across marine, freshwater, and terrestrial ecosystems [12]. It has been suggested that these two elements would quickly disappear from the marine environment in the event of a cessation of chitin degradation [12]. Chitinases (EC 3.2.1.14) are hydrolytic enzymes that catalyze the degradation of chitin by breaking down the O-glycosidic bonds in chitin chains, producing chitooligosaccharides [13]. Natural chitin‘s insolubility to aqueous dissolution is due to the presence of intermolecular hydrogen bonds in its linear macromolecules, which enhance its structural stability and hydrophobicity [14,15]. This property imposes critical constraints on potential chitin applications. Nevertheless, as the exclusive alkaline amino oligosaccharides with positively charged ions in nature, chitooligosaccharides possess several advantageous properties, including high solubility, biocompatibility, and bioactivity [16]. Therefore, the identification of chitinases is of great significance not only for understanding the role of chitin degradation in the carbon and nitrogen cycles, but also for the promotion of the high-value application of chitin derivatives.

Chitinases are formally classified into endochitinases (EC 3.2.1.14) and exochitinases (EC 3.2.1.200/201) based on their catalytic mechanisms by the International Union of Biochemistry and Molecular Biology. Endochitinases (EC 3.2.1.14) hydrolyze β-1,4-glycosidic bonds within chitin and chitodextrins through a non-processive, stochastic cleavage mechanism, generating heterogeneous chitooligosaccharides [e.g., (GlcNAc)_2–6_] and exposing free chain termini. These newly accessible termini serve as substrates for exo-acting enzymes: exochitinases (EC 3.2.1.200) and exochitodextrinases (EC 3.2.1.201), which processively hydrolyze *N*,*N’*-diacetylchitobiose from either the non-reducing (EC 3.2.1.200) or reducing (EC 3.2.1.201) ends, respectively [17]. In contrast with cellulose degradation, a modest number of enzyme families are involved in chitinolysis [18] and are classified into GH18, GH19, and GH20 in the CAZy database [6,19]. Chitinases belonging to the GH18 and GH19 families catalyze the degradation of chitin polymers [20]. The GH18 chitinases are ubiquitous in bacterial species, whereas the GH19 chitinases are predominantly found in plants and are postulated to function as a defense mechanism against fungal pathogens [21,22]. The chitinase GH19 family members are involved in a range of physiological processes, including pathogenesis, nutrition, growth regulation, and immunity [23,24]. Previous studies have primarily concentrated on the functional characterization [25], structure [26], genome, and transcriptome analysis [27] of GH18 chitinases, but there were few studies on GH19 family chitinases [28], and even fewer studies on them in diatoms.

Given the abundance of chitin reserves and the extensive biotechnological applications of its derivatives, a lot of research has focused on the high-value potential of chitin. In this study, we investigated chitinase gene families in *Thalassiosira weissflogii* by retrieving its genomic dataset. Our analysis identified 38 putative chitinase genes and analyzed their sequence structural features, phylogenetic relationships, homology comparisons, and subcellular locations at the transcriptional and enzymatic levels. Since diatom-derived GH19 chitinases have not been characterized, we heterologously expressed and functionally validated one chitinase (TwChi2) from the GH19 family. TwChi2 has been proved to have high thermal stability and to be a novel exochitinase (EC 3.2.1.201) catalyzing the degradation of both chitin oligomers and polymers. This is the first report on the functional properties of GH19 chitinase in diatoms. The findings of our study offer insights into the process of β-chitin degradation in diatoms and provide a reference for the high-value utilization of different diatom-derived chitinases.

## 2. Results

### 2.1. Identification and Analysis of Chitinase Family Genes in T. weissflogii

A total of 38 putative chitinase genes were identified in the transcriptome of *T. weissflogii* (Table S1). Detailed molecular characteristics including gene names, gene IDs, nucleotide and amino acid sequence lengths, molecular weights (MW), and theoretical isoelectric points (pIs) are presented in Table 1. The sequence lengths of the chitinases ranged from 218 (TwChi6) to 3610 (TwChi38) amino acid (AA) residues, with an average length of 1199 AA. The relative molecular mass varied from34.34 kDa (TwChi6) to 390.98 kDa (TwChi38), and the pI values ranged from 3.61 (TwChi34) to 9.19 (TwChi36), reflecting diverse functional potentials within this enzyme family.

**Figure 1:**
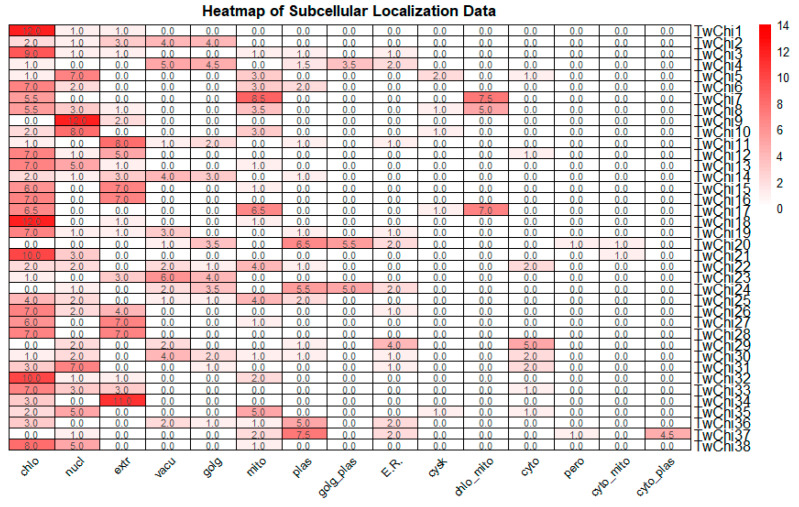
A heat-map of subcellular locations of chitinase proteins in *Thalassiosira weissflogii*. chlo: chloroplast; nucl: nuclear; extr: extracellular; vacu: vacuolar; golg: golgi apparatus; mito: mitochondrion; plas: plasma membrane; golg_plas: golgi apparatus plasma membrane; E.R.: Endoplasmic reticulum; cysk: cytoskeleton; chlo_mito: chloroplast_mitochondrion; cyto: cytoplasm; pero: peroxisome; cyto_mito: cytoplasm_mitochondrion; cyto_plas: cytoplasm_plasma membrane.

Table S2 summarizes computational predictions of signal peptides, transmembrane helices (TMHs), and subcellular localizations for the *T. weissflogii* chitinases. SignalP-5.0 predictions indicate that 68.42% of them (26 out of 38) possessed *N*-terminal signal peptides, suggesting that these chitinases are involved in the secretory pathway. Eleven (TwChi1, TwChi5, TwChi7, TwChi8, TwChi10, TwChi19, TwChi20, TwChi29, TwChi31, TwChi35, and TwChi36) were predicted by HECTAR to have type II signal anchors, indicating that they may be Type II transmembrane proteins with an *N*-terminal cytoplasmic domain and may be localized on chloroplast or mitochondrial membranes. Nine (TwChi4, TwChi6, TwChi9, TwChi19, TwChi20, TwChi22, TwChi23, TwChi25, TwChi36) were predicted to contain TMHs, with three overlapping the type II signal anchor group (TwChi19, TwChi20, TwChi36), suggesting a subcellular localization at the plasma membrane or the endomembrane system. Additionally, the results by WoLF PSORT and HECTAR regarding subcellular locations indicated that most TwChi proteins are located in the chloroplast (44.74%) and a few are present in the nucleus (13.16%) and extracellularly (7.89%) (Table S2, Figure 1). There was also great variation among TwChi genes regarding subcellular locations.

### 2.2. Phylogenetic Analysis, Gene Structures, Motifs, and Conserved Domains of the TwChis

**Figure 2:**
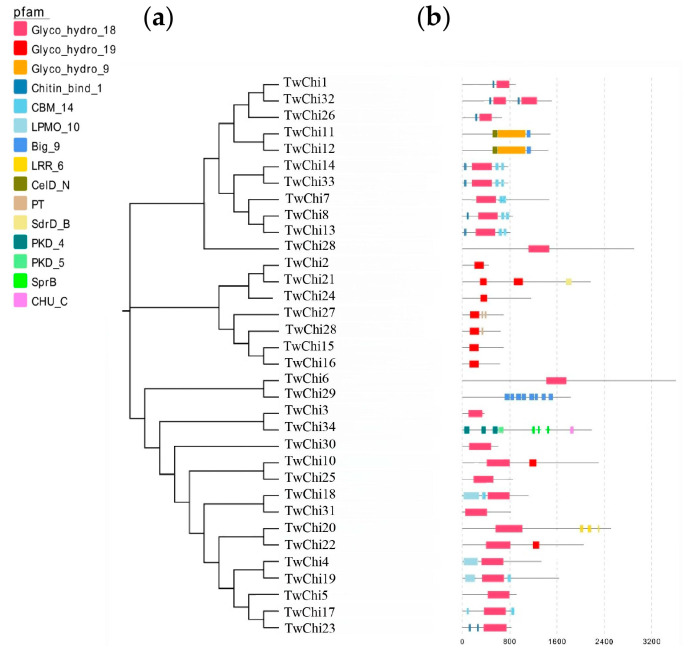
Phylogenetic relationships, analysis of domains, and conserved motifs of the *T. weissflogii* chitinases. (**a**) Phylogenetic tree of 34 *T. weissflogii* chitinase proteins constructed with MEGA using the NJ (Neighbor-Joining) method and 1000 bootstrap replicates. (**b**) Gene domain distribution of the *T. weissflogii* chitinases. The full names of domain abbreviations are listed in Table S13. The lengths of domains can be inferred by the scale at the bottom.

An unrooted phylogenetic tree was constructed to reveal distinct evolutionary relationships among the chitinases of *T. weissflogii*. A total of 38 protein sequences from the *T. weissflogii* were used to build the phylogenetic tree (Figure 2) and multiple sequence homology alignments were performed. The results revealed that 38 sequences were in the range of 70–250 AA, with a high degree (50%) of sequence identity (Figure S1). The sequence differences within the chitinase family were further analyzed by examining their conserved domains and motifs using the Pfam database (Figure 2) and the MEME program (Figure S2), respectively. Twenty conserved motifs (15–65 AA) were identified in the *T. weissflogii* chitinase proteins (Table S3). Domain architecture analysis identified three glycoside hydrolase families: Glyco_hydro_18 domain (*n* = 23), Glyco_hydro_19 domain (*n* = 9), and Glyco_hydro_9 domain (*n* = 2), with TwChi10/22 displaying unique GH18-GH19 domain fusions. Notably, nine other auxiliary domains, Chitin_bind_1 (pfam00187), CBM_14 (Peritrophin A, pfam01607), LPMO_10 (pfam03067), CHU_C (pfam13585), PKD (cd00146), LRR_6 (pfam13516), CelD_N (pfam02927), SdrD_B (pfam17210), and SprB (pfam13573), were detected in 18 protein sequences (47.37%). Two TwChi (TwChi29 and TwChi34), derived from the genome annotation pipeline, do not contain Glyco_hydro domains but have chitinase auxiliary domains (CBM96 and PKD). So, we consider that they are chitinase-related proteins rather than canonical chitinases, pending experimental confirmation. In addition, three motif combinations corresponding to the two Glyco_hydro domains were identified. The combination of motifs 4, 2, 1, and 18 was detected in the Glyco_hydro_19 domain, which was identified in chitinases TwChi21, TwChi2, TwChi24, TwChi27, TwChi28, TwChi15, TwChi16, and TwChi22. Two other highly conserved motif combinations were identified in the Glyco_hydro_18 domain. Chitinases TwChi3, TwChi14, TwChi33, TwChi7, TwChi8, TwChi9, and TwChi6 contained the combination of motifs 14, 20, 1, 9, 11 and 6, whereas TwChi30, TwChi10, TwChi25, TwChi18, TwChi31, TwChi20, and TwChi23 contained the combination of motifs 3, 8, 17, and 5. However, five chitinases (TwChi30/35/36/38) lacked identifiable motifs, suggesting divergent evolutionary trajectories. In general, chitinases clustered in the adjacent branches have similar motifs and domain compositions.

Three main types of secondary structure elements (α-helix, extended strand, and random coil) were predicted in the structural models of the *T. weissglogii* chitinases (Table S4). The random coil was the predominant component of the chitinase secondary structure, accounting for an average of 69.17%. α-helix constituted 17.51%, extended strand constituted 14.22% of the secondary structures and β-turn constituted 0%. Three-dimensional protein models have been constructed for the *T. weissglogii* chitinases to facilitate a deeper understanding of their structural characteristics (Table S5). The 3D models of *T. weissglogii* chitinases were compared to known experimental structures deposited in the SWISS-MODEL database, and 88.89% of the hits were chitin-degrading enzymes from a wide range of phylogenetic origins, including viruses (TwChi18), bacteria (TwChi1, TwChi3, TwChi4, TwChi5, TwChi6, TwChi7, TwChi9, TwChi10, TwChi20, TwChi22, TwChi23, TwChi26, TwChi30, TwChi32), plants (TwChi15, TwChi16, TwChi24, TwChi37), insects (TwChi25), and humans (TwChi13, TwChi14, TwChi17, TwChi19, TwChi31, TwChi33), suggesting conserved catalytic mechanisms across phylogenetically distant species.

### 2.3. Sequence Analysis of TwChi2

Detailed analysis and characterization focused on TwChi2 (mikado.scaffold_10G336.1), a GH19 chitinase (Pfam: PF00182) containing a single catalytic domain (Glyco_hydro_19). The sequence features of TwChi2 are summarized in Table 2. TwChi2 (1362 bp) is encoded by 453 amino acids and has a predicted molecular weight (MW) of 50.39 kDa and an isoelectric point (pI) of 5.03. Secondary structure prediction shows that TwChi2 had a predominance of the α-helix structure (~15%).

**Figure 3:**
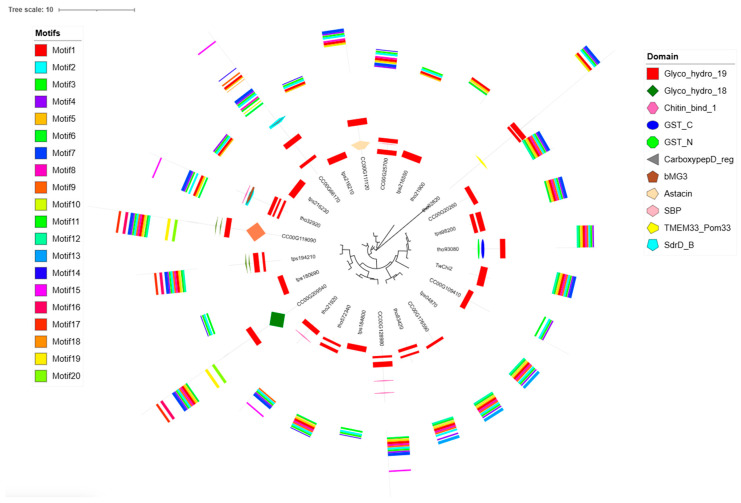
Phylogenetic relationships and domain architectures of the 25 chitinases (Chis) from *Thalassiosira oceanica* (To), *Thalassiosira pseudonana* (Tp), *Cyclotella cryptica,* and *Thalassiosira weissflogii* (Tw). Inner layer: an unrooted phylogenetic tree constructed with MEGA using the ML (Maximum Likelihood) method. Middle layer: Distribution of the chitinase protein domains, denoted by boxes of different colors and shapes (rectangle: Glyco_hydro_19; rhombus: Glyco_hydro_18; horizontal hexagon: Chitin_bind_1; ellipse: GST_C; octagon: GST_N; left pointing triangle: CarboxypepD_reg; up pointing pentagram: bMG3; right pointing pentagram: Astacin; vertical hexagon: SBP; left pointing pentagram: TMEM33_Pom33; down pointing pentagram: SdrD_B). Outer layer: Distribution of the chitinase twenty putative motifs, indicated with different colored boxes. Table S8 presents details of the motifs.

For the phylogenetic analysis, 24 diatom sequences highly homologous to the full-length amino acid sequence of TwChi2 were retrieved from the three available genome resources (*T. pseudonana*, *T. oceanica*, *C. cryptica*) of PLAZA databases (Tables S7 and S8). These sequences were highly enriched in Thalassiosirales species (62.5%), reflecting order-specific gene expansion. In phylogenetic trees constructed with TwChi, highly homologous sequences, almost all chitinase sequences (92%), have the domain Glyco_hydro_19 (except tho21900 and tps04870). Interestingly, the *C. cryptica* homolog CC00G209540 contained both the Glyco_hydro_18 and the Glyco_hydro_19 catalytic domains. The combination of motifs 12, 6, 18, 10, 5, 1, 8, 9, 2, 3, and 4 was detected in the Glyco_hydro_19 domain, which was identified in chitinases TwChi2, tps194210, CC00G20260, tps98200, CC00G176590, tps04870, tho83420, CC00G209540, and tho93080. In general, chitinases in the same branch had similar motif compositions (Figure 3, Table S8). In addition, three endochitinase and five exochitinase were retrieved from the NCBI databases (Table S10). TwChi2 and exochitinase were found to cluster together, suggesting that TwChi2 may have exochitinase activity (Figure S3).

### 2.4. Heterologous Expression and Chitinase Activity of TwChi2

**Figure 4:**
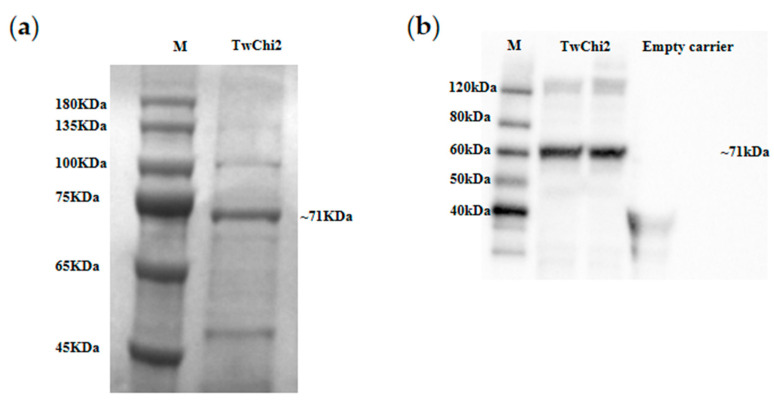
SDS-PAGE (**a**) and Western blot (**b**) analysis of recombinant TwChi2. M: protein ladder. To improves the clarity and conciseness of the presentation, these two gels have been cropped. Original SDS-PAGE and Western blot are shown in the Figure S3 (Supplementary Information). The fusion protein (~71 KDa) includes TwChi2 (50.4 KDa), Trx·Tag™ (≈12 kDa), His·Tag (≈0.8 kDa), S·Tag™ (≈1.6 kDa), and linker sequences (≈2 kDa).

To functionally characterize TwChi2, we induced large amounts of its expression within *E. coli* heterologous cells. Affinity chromatography yielded 3.12 mg/mL of purified TwChi2 with an apparent molecular weight of approximately 71 kDa, consistent with theoretical predictions (50.4 kDa native protein + 20.8 kDa fusion partner). The Western blot analysis using anti-His antibodies confirmed the recombinant protein’s integrity, confirming the presence of protein with the expected size (Figure 4 and Figure S4).

**Figure 5:**
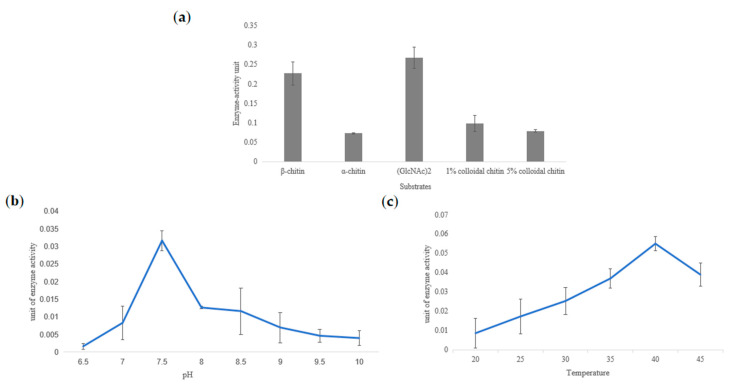
Chitinolytic activity of TwChi2. (**a**) Enzymatic activities of TwChi2 with β-chitin, α-chitin, (GlcNAc)_2_, and 1% and 5% α-colloidal chitin as substrates. (**b**) Influence of pH (6.5–10) on the activities of TwChi2 with (GlcNAc)_2_ as substrate. (**c**) Influence of temperature (20–45 °C) on the activities of TwChi2 with (GlcNAc)_2_ as substrate. The error bars are the mean ± SD of three technical replicates for each sample.

**Figure 6:**
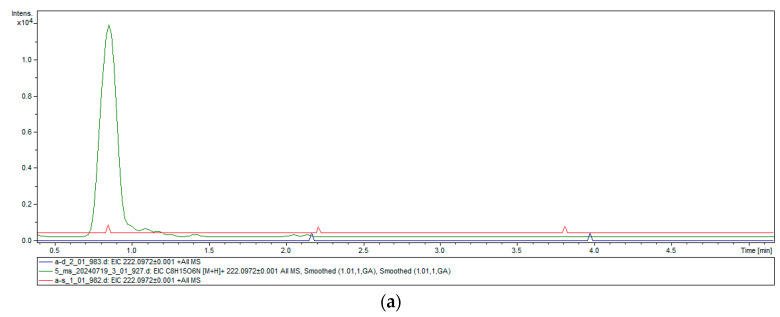

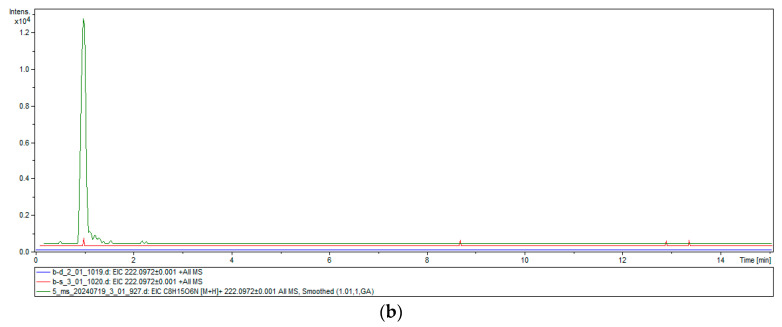
UPLC-Q-TOF-MS-MS chromatograms. Purified TwChi2 and α-chitin (**a**) and β-chitin (**b**) were taken as the experimental group (red curves), and inactivated purified TwChi2 (blue curves) and GlcNAc aqueous solution (green curves) were taken as the control group, respectively. The characteristic peak appeared at about 0.8 min in the experimental group, with the predicted molecular weight of 222.0972 ± 0.001, which is in line with the MW of chitin monomer *N*-acetylglucosamine. Note the molecular ion species formed: [M + H]^+^.

The chitinase activities of TwChi2 on (GlcNAc)_2_, colloidal chitin, α-chitin, and β-chitin were detected by using the DNS method (Figure 5a) and UPLC-Q-TOF-MS-MS (Figure 6 and Figure S2). The DNS assay showed reducing sugar production across all of the five experimental groups (TwChi against biopolymer, 1% and 5% colloidal chitin, α-chitin, and β-chitin). Taking the amount of enzyme required to produce 0.1 μmol GlcNAc in 1 h as the unit of enzyme activity, the enzyme activity was measured and a bar chart with the number of units of enzyme activity per milligram of enzyme was prepared, as shown in Figure 5a. We found that TwChi2 had higher enzyme activity when biopolymer and β-chitin were used as substrates. We compared the difference in OD540 between the experimental group and the control group. The results indicate that regardless of whether β-chitin or (GlcNAc)_2_ was used as the substrate, TwChi2 showed significantly higher chitinolytic activity than the control groups, with the activity being 1.5-fold (against biopolymer) and 1.17-fold (against β-chitin) higher than the control groups (*p* < 0.05), respectively (Figure 5a). It was found that TwChi2 had the highest chitinolytic activity when (GlcNAc)_2_ was used as the substrate. We then detected its chitinolytic activity at different temperatures and pH levels with (GlcNAc)_2_ as the substrate. The results of the enzymatic characterization illustrate that TwChi2 has the highest catalytic activity at 40 °C (Figure 5b) and pH 7.5 (Figure 5c). All of the source data of the enzyme activity under different substrates and different temperatures are shown in Tables S11–S13.

In addition, in order to detect whether TwChi has exochitinase activity or endochitinase activity, we employed the UPLC-Q-TOF-MS-MS method to profile the molecular weight of the hydrolysis products of TwChi with β-chitin and α-chitin, with the inactivated TwChi solution as a control. A clear characteristic peak appeared at about 1 min in the experimental group, which was consistent with the peak time of the GlcNAc standard substance, with a predicted molecular weight of 222.0972, which is in line with the MW of chitin monomer *N*-acetylglucosamine (Figure 6). High-resolution MS analysis confirmed the molecular identity ([M + H]^+^ = 222.0972; theoretical 222.0974), unambiguously demonstrating GlcNAc monomer production, confirming exochitinase (EC 3.2.1.201) activity.

## 3. Discussion

Chitin represents the predominant renewable polymer in marine ecosystems, and diatoms are the major producers of β-chitin. β-chitin exhibits superior physicochemical properties compared to α-chitin, particularly in solubility and enzymatic accessibility. Chitinase can catalyze chitin to produce more valuable chitin oligosaccharides with demonstrated pharmaceutical potential. The functional verification of chitinase from marine β-chitin producers remains unexplored, although the chitinase gene family has been extensively studied in insects, higher plants, and fungi [18,20,21,29]. Previously, we performed sequencing and assembly of the *T. weissflogii* genome (not yet published), making it possible to identify its putative chitinase genes. Compared with the GH18 hydrolase family, chitinases belonging to GH19 have rarely been reported and even fewer have been investigated regarding the function of chitinases from β-chitin resources. In this study, chitinase genes were retrieved from the genome sequences of *T. weissglogii*, and their sequence structure, phylogenetic relationship, subcellular localization, and enzyme activity were investigated. This is the first comprehensive report of GH19 chitinases in diatoms, paving the way for improving the in vitro application of chitinase.

### 3.1. Diverse Structure and Subcellular Locations of Chitinases in T. weissflogii Showed Their Various Biological Function

The *T. weissflogii* genome systematically identified 38 chitinase homologs through conserved domain analysis, exceeding chitinase genes of *T. pseudonana* (24 chitinase-encoding genes) [10] and *Cyclotella cryptica* (22 chitinases) [30]. Members of the *T. weissflogii* chitinases exhibit distinct predicted 3D structural conformations (Table S5), indicating that they may be involved in a range of biological processes, including stress-related responses, growth, and developmental operations [10].

Chitin fibrils are located between the silica frustule and the cell membrane of chitin-producing diatoms [31]. However, the location of chitin degradation sites remains unknown in diatoms. We investigated the subcellular locations of the TwChi proteins that were primarily interpreted based on the results of WoLF PSORT and HECTAR. The HECTAR predictions indicate that approximately half of the chitinase family members are involved in the secretory pathway, which is consistent with the finding that the majority of chitinase genes in *Brassica rapa* and *Arabidopsis thaliana* are secretory [32,33]. This suggests that chitinases have extracellular activity and important in vitro applications. The complex cellular compartmentalization predicted for chitinases in *T. weissflogii* indicates that they may perform specific functions more efficiently in various biological processes, which is in accordance with their diverse 3D structures. Notably, all these probable subcellular locations were from software predictions and further protein localization experiments, and methods such as the introduction of fluorescent tags are necessary to confirm our predictions.

### 3.2. TwChis Principally Belong to the Class II GH19 Family with More CBMs

The GH19 family of chitinases is found in more plants, yet it is common in diatoms. The constructed phylogenetic tree demonstrates that the GH19 chitinases clustered on the same branch. This phenomenon was also observed in *T. pseudonana* and *T. oceanica* [10], which indicates that the GH19 chitinase is a significant component of chitinase in diatoms and may play a crucial role in chitin degradation in diatoms.

Sequence-based classifiers group plant chitinases into six distinct classes [34], where GH19 encompasses Classes I, II, and IV. The N-terminal cysteine-rich domain of Classes I and IV is involved in chitinous binding, which lacks in Class II chitinases. In contrast to Class I and II chitinases, the catalytic domain of class IV chitinases has undergone multiple amino acid deletions at two distinct locations [31]. In this study, we found that *T. weissflogii* contains GH19 chitinases, and most of them are Class II chitinases with the absence of a cysteine-rich domain in the N-terminus, which is different from other species that mostly contain GH18 chitinase [35]. Furthermore, the conserved motifs of the sequences were identified. The results demonstrate that the Glyco_hydro_19 domain motifs are invariably conserved.

Chitinases typically exhibit modular architectures containing a catalytic domain and additional auxiliary domains, such as carbohydrate-binding modules (CBMs) [36]. These are classified into 106 families in the CAZy database (http://www.cazy.org/ (accessed on 15 February 2025)). CBMs facilitate the enzyme’s interaction with the substrates, disrupting its crystalline structure and generating free chain ends, thereby enhancing the catalytic activity of the enzyme against insoluble substrates [36,37]. In the present study, the CBM_ 14, Chitin_bind_1, CelD_N, and LMPO_10 domains, which are typically found in chitin-binding proteins, were identified in the protein sequences of 39.5% chitinase, five of which possessed two or more CBM_14 domains (Figure 1). This finding indicates that *T. weissflogii* chitinases have the capacity of binding chitin. Moreover, eight chitinases contained the Chitin_bind_1 domain, and none of these belong to the GH19 family. This indicates that the chitin-binding domain is non-essential for the GH19 family to perform its functions. Subsequent experiments yielded results that corroborated this hypothesis.

### 3.3. TwChi2 Was Proved to Be a Novel Exochitinase Catalyzing Chitin Polymer Degradation

Considering the fewer investigations on the function of GH19 in diatoms, we prioritized TwChi2 as the candidate for further heterologous expression and enzymatic determination. Homology searches against the PLAZA 5.0 database identified conserved chitinolytic systems in marine diatoms (*C. cryptica*, *T. oceanica,* and *T. pseudonana*), indicating that marine diatoms contain abundant Chi homologs. Phylogenetic tree analysis showed that TwChi2 was closely related to tps98200, tho93080, and CC00G20260, with similar domains and conserved motifs. This may be due to the fact that *T. weissflogii* belongs to Thalassiosiraceae with *T*. *oceanica* and *T. pseudonana*. The evolutionary position of *C. cryptica* is adjacent to *T. pseudonana*, which is consistent with the finding that *T. pseudonana* likely descended from a freshwater ancestor in *Cyclotella* [38].

We found that TwChi2 had optimum chitinolytic enzyme activity at 40 °C. At present, the optimal conditions for the enzyme activity of chitinase have been studied in animals, plants, bacteria, and fungi. According to the previous results, the optimal temperature was 18–90 °C [36,37,39,40]. The optimum temperature of 40 °C measured in our study is within that range, which is consistent with the optimum temperature of Chis from *Oryza sativa* (40 °C) and *Vibrio parahaemolyticus* (40 °C) [41,42]. The high optimum temperature of TwChi2 is likely due to the high thermal stability of chitinase in *T. weissflogii*. This phenomenon does not appear to be associated with the optimal temperature range for the growth and survival of the algae. The majority of chitinases that have been characterized to date exhibit thermal stability [43]. Sekiguchi et al. (1995) found that optimum observed temperature of Chi was 60 °C in both *Chondrus giganteus* and *Gigartina mikamii* [44]. The results also showed that the optimum pH of TwChi2 was 7.5, which is close to the environmental habitat of *T. weissflogii*.

Previous enzymatic characterizations of chitinases have predominantly utilized colloidal chitin or soluble chitooligosaccharides as preferred substrates [43,45,46]. Conversely, chitin polymer has been used as substrates in only a small number of studies [11]. Additionally, some studies have reported that GH19 chitinase is predominantly active on soluble chitin oligomers while the GH18 enzymes markedly preferred crystalline chitin forms [44]. It is also reported that GH18 chitinases play a major role in the degradation of various chitin forms while the GH19 enzyme could play a minor role in degradation or may even be dispensable [35]. Zhu et al. (2008) found that differences in the types of chitin (α, β, or γ) may require different chitinases for the effective turnover of chitin [47]. However, our results contradict these perspectives. The GH19 chitinase TwChi2 in *T. weissflogii* exhibits catalytic activity not only on colloidal chitin and chitooligosaccharides but also on insoluble α-chitin and β-chitin. This broad specificity may result from the structural adaptation of its catalytic cleavage, i.e., the ability of the enzyme to regulate substrate binding through dynamic conformation [48], enabling dynamic adaptation to diverse substrate geometries. This original finding provides a good candidate chitinase, catalyzing the production of more valuable chitin oligomers.

Chitinolytic mechanisms are categorized into two mechanistic classes: endochitinases (EC 3.2.1.14), which cleave the chitin polymer at random internal sites, and exochitinases, which degrade the chitin chain from the nonreducing end (EC 3.2.1.200) or the reducing end (EC 3.2.1.201) [49,50]. A chitin hydrolase with exochitinase activity therefore has significant potential for the production of GlcNAc by enzymatic methods. Given the detection of GlcNAc monosaccharides (m/z 221.092) in the reaction mixture by UPLC-Q-TOF-MS-MS, it may be posited that TwChi2 exhibits exochitinase (EC 3.2.1.201) activity. This renders TwChi2 a significant candidate enzyme in the production of GlcNAc monomers. However, no dimers were identified in the reaction products, indicating that TwChi2 does not possess endochitinase activity.

## 4. Materials and Methods

### 4.1. Sample Culture and Treatment

*Thalassiosira weissflogii* (9021) was acquired from the Microalgae Collection Center at Ningbo University, Ningbo, China, and grown in optimized f/2 liquid medium provided by Shanghai Guangyu Biological Technology Co., Ltd., Shanghai, China [10]. Cells were cultured at 20 °C with 12 h:12 h light–dark cycles (100 µmol m^−2^ s^−1^) at 100 rpm. Cells were collected by centrifugation at the exponential growth stage, flash-frozen in liquid nitrogen, and stored at −80 °C for further experiments.

### 4.2. Identification of Chitinase Genes in T. weissflogii

The nucleotide sequences and corresponding protein sequences of *T. weissflogii* chitinase genes were retrieved from the genome data (NCBI: SRR32271522) of *T. weissflogii* using “chitinase” and other relevant keywords, e.g., “chitin hydrolase”, “chitin degradation”, and “family 19 glycoside hydrolase” listed in Table S14. A total of 38 hits were annotated as chitinase-relevant genes, which were designated as TwChis in our study.

### 4.3. Sequence Analysis and Structural Characterization

All of the nucleotide sequences were analyzed using the ExPASy ProtParam tool (https://web.expasy.org/protparam/, accessed on 27 October 2024) to calculate their molecular weights and pIs [51]. MEME V5.1.1 online software was used to identify conserved motifs in the chitinase proteins with the following parameters: any number of repetitions, maximum of 20 motifs, and an optimum motif width of 6–200 amino acids [52]. The Motif search tool (http://www.genome.jp/tools/motif/, accessed on 27 October 2024) was applied to analyze the domain in the Pfam and NCBI-CDD databases. WoLF PSORT and HECTAR were jointly used to predict the TwChis subcellular localization [13,53], with the SignalP v4.1 server (https://services.healthtech.dtu.dk/services/SignalP-4.1/, accessed on 27 October 2024) for signal peptide and signal anchoring sequence prediction [54]. TMHMM Server v2.0 (https://services.healthtech.dtu.dk/services/TMHMM-2.0/, accessed on 27 October 2024) was used to predict transmembrane helices. The secondary structures were illustrated by ESPript [55], and the tertiary structure was predicted by SWISS-MODEL [56]. Multiple sequence alignment of all predicted chitinase proteins was performed with the MUSCLE method [57]. Next, MEGA X was used to construct the Neighbor-Joining tree with 1000 bootstrap replicates [58], a p-distance substitution model, and pairwise deletion gaps parameters.

### 4.4. Cloning and Sequence Analysis of TwChi

Total RNA was extracted by grinding the *T. weissflflogii* frozen sample in TRIzol reagent (Life Technologies, Carlsbad, CA, USA), and then the extracted RNA was reverse transcribed into cDNA using a reverse transcription kit (SparkJade, Jinan, China). The synthesized cDNA could be directly used for subsequent PCR amplification.

A full-length chitinase homologous sequence with Glyco_hydro_19 (PF00182) (mikado.scaffold_10G336.1, TwChi2) was retrieved from the *T. weissflogii* genome dataset. The full-length *TwChi2* gene was synthesized with codons optimized for *E. coli* (Sangon Biotech, Shanghai, China). TwChi2-F (5′-CGGGATCCATGCCGCCCACCAAATCAATTC-3′) and TwChi2-R (5′-CCAAGCTTTCCAATCCTTATCCGCCCAAGA-3′), containing an *Bam*HI and a *Hin*dIII restriction site, respectively, were designed for PCR amplification. A total of 20 μL of PCR reaction mixture was used, with 1 μL of each primer, 10 μL of 2 × Taq Master Mix (Vazyme, Nanjing, China), 2 μL of cDNA template, and 6 μL of ddH_2_O. The PCR amplification procedure was as follows: 95 °C for 30 s; 35 cycles of 95 °C for 10 s, 55 °C for 5 s, and 72 °C for 1 min 30 s; and 72 °C for 1 min. The resulting products were sequenced and analyzed using BLASTn + 2.16.0.

### 4.5. Phylogenetic Analysis of TwChi2 Homologous Sequences in Diatoms

For phylogenetic analysis, sequences highly homologous to the full-length amino acid sequence of TwChis were retrieved from PLAZA (https://bioinformatics.psb.ugent.be/plaza/versions/plaza_diatoms_01/, accessed on 20 October 2024). Homology alignment was conducted using BLASTP + 2.16.0. MEGA X was used to construct the Maximum Likelihood tree based on the WAG with the Fregs. (+F) model and Gamma Distributed (G) matrix-based model [59].

### 4.6. Expression and Purification of Recombinant TwChi2

The pET system with His-tag (Shanghai Sangon, Shanghai, China) was used to express TwChi2 in vitro. The plasmids TOPO-TwChi2 and pET-32a were extracted according to the steps of the TaKaRa MiniBEST Plasma Purification Kit (Takara, Japan). The extracted plasmids were digested with HindIII and EcoRI and then linked by T4 ligase, resulting in the fusion plasmid pET-32a-TwChi2. The Escherichia coli strain BL21 plysS was transformed with the recombinant expression plasmid pET-32a-TwChi2. The transformed *E. coli* cells were then incubated in 1 L of Luria–Bertani (LB) medium with 100 µg·mL^−1^ of ampicillin at 37 °C. When OD600 reached 0.6, 0.1 mM IPTG was supplemented, and the induction conditions were changed to 15 °C and 120 rpm. After 24 h of incubation, the transformed cells were harvested by centrifugation at 4500 rpm at 4 °C for 30 min. The cell pellets were resuspended in a buffer containing 20 mM sodium phosphate, 500 mM NaCl, 5% glycerol, and 20 mM imidazole buffer at pH 8.0. One EDTA-free protease inhibitor tablet (Roche, Basel, Switzerland) was added to the 35 mL extracts prior to the ultrasonication. Then, the cells were disrupted by sonication for 25 min (3 s on and 5 s off cycle) (Xinzhi, Ningbo, China) and cell debris was removed by centrifugation at 12,000 rpm, 4 °C for 45 min. The supernatant was then filtered through a 0.45 μm filter membrane to obtain crude enzyme solution.

The overexpressed TwChi2 was purified by a chromatographic step using the ÄKTA Pure system (GE Healthcare, Fairfield, CA, USA) equipped with a His HP (GE Healthcare, Fairfield, UK). The column was equilibrated with 50 mL (10 column volumes) of buffer A (20 mM sodium phosphate, 20 mM imidazole, 500 mM NaCl, and 5% glycerin; pH 8.0) at a flow rate of 5 mL·min^−1^. Then, 150 mL of the sample (bacterial extract diluted 5 times by buffer A) was injected at a rate of 1.2 mL·min^−1^. The non-adherent proteins were removed by rinsing with 20 volumes of buffer A. The protein was eluted by a gradient increase in the proportion of buffer B (20 mM sodium phosphate, 500 mM imidazole, 500 mM NaCl, and 5% glycerin; pH 8.0) at a rate of 3 mL·min^−1^. The eluates were collected by the collector to obtain the purified protein.

### 4.7. SDS-PAGE and Western Blotting

The protein concentration in the purified elutes was determined with a BCA kit (Solarbio, Beijing, China), according to the manufacturer’s instructions. We then boiled the samples for 10 min in loading dye (125 mM Tris-HCl, 10% SDS, 0.25% BPB, 10% 2-Mercaptoethanol, 50% Glycerol) and loaded 60 μg of the crude extract onto 12% acrylamide gels for separation by SDS-PAGE. Primary (Anti His-Tag Mouse Monoclonal Antibody at a concentration of 1:500) and secondary (Goat Anti-Mouse IgG, HRP conjugated at a concentration of 1:2000) antibody were used for a Western blot test performed at room temperature for 4 h using the iBind™Flex Western System (Thermo, Waltham, MA, USA), following the manufacturer’s protocol. After washing with ddH2O solution three times, TwChi2 target bands were visualized by incubating the membranes in ECL regents with a ChemiDoc XRS Imaging System and Image Lab 6.0 software (Bio-Rad Lab, Inc., Hercules, CA, USA).

### 4.8. Chitinase Activity Assay

Chitinolytic activity was estimated by using a dinitrosalicylic acid (DNS) method using α-chitin, colloidal α-chitin, β-chitin, and chitin dimer (GlcNAc)_2_ as substrates, according to the method described by Mohun et al. (1962) with modifications [60]. The reaction matrix had 0.5 mL of 2 mg/mL chitin or (GlcNAc)_2_ suspension in a 20 mM sodium phosphate buffer at pH 7.5, in 0.5 mL of enzyme solution. The inactivated purified enzyme was used as the control group, and the denatured protein precipitate was removed by centrifugation. The mixture then incubated 60 min at 37 °C and then the reaction was terminated by 0.75 mL DNS (NaOH 10 g/L, dinitrosalicylic acid C_7_H_4_N_2_O_7_ 10 g/L, phenol C_6_H_6_O_2_ g/L, and adding Na_2_SO_3_ 0.05 g/100 mL and sodium potassium tartrate C_4_H_4_KNaO_6_·4H_2_O 20 g/100 mL when using). The chromogenic reaction of the mixture was developed by incubating it for 10 min at 98 °C. Centrifugation was performed at 7500× *g* for 10 min, then the supernatant absorption was measured at 540 nm. OD540 was detected in a 96-well plate using a Biotek EON microplate reader (Biotek, Santa Clara, CA, USA). A standard curve was plotted using *N*-acetyl glucosamine (NAG, Sigma, St. Louis, MO, USA). One unit (U) of chitinase activity represents the amount of enzyme required to produce 0.1 μmol GlcNAc in 1 h under reaction conditions. To obtain the optimal reaction conditions for the TwChi2, we measured the enzyme activity at various temperatures (20, 25, 30, 35, 40, and 45 °C)and different pH values (6.5, 7.0, 7.5, 8.0, 8.5, 9.0, 9.5 and 10.0). Each experiment was performed in triplicate, and a statistical analysis was conducted with SPSS 26.0 (SPSS Inc., Chicago, IL, USA). The chemical formula prediction of the reaction products was detected by ultra-high-performance liquid chromatography combined with time-of-flight mass spectrometry (UPLC-Q-TOF-MS-MS), with the inactivated purified enzyme group as the control groups. Semipreparative HPLC separation was performed on a Waters Agilent 1260 instrument equipped with a Poroshell 120 EC-C18 column (50 mm × 3.0mm, 2.7 μm, Agilent, Santa Clara, CA, USA). HR-ESI-MS spectra were obtained using a Q-TOF-MS instrument (Bruker Maxis plus, Billerica, MA, USA).

## 5. Conclusions

In this study, we systematically identified 38 TwChi genes from the *T. weissflogii* genome, characterized their phylogenetic relationship, conserved motifs and structural domains, predicted subcellular localization patterns, conducted comparative analysis of GH19 chitinase bioinformatics analysis, and experimentally validated enzymatic functions via heterologous expression profiling coupled with biochemical optimization of catalytic parameters. The results showed that GH19 chitinase TwChi2 from *T. weissflogii* is a member of exochitinase (EC 3.2.1.201) with strong thermal stability and can hydrolyze biopolymer, colloidal α-chitin, α-chitin and β-chitin. Our report contributes to a more profound comprehension of the metabolic processes involved in chitin metabolism in diatoms, and provides a candidate enzyme for the environmentally friendly industrial preparation of chitin oligosaccharides.

## Figures and Tables

**Table 1:** Sequence information of 38 TwChis in terms of gene/protein names, gene IDs, nucleotide and amino acid sequence lengths, molecular weights (MW), and theoretical isoelectric points (pIs). CDS, coding sequences.

Name	Gene ID	Length of CDS (bp)	Number of Amino Acids	Molecular Weight (KDa)	Isoelectric Point (pI)
TwChi1	mikado.scaffold_31G129.1	2703	900	95.71	4.76
TwChi2	mikado.scaffold_10G336.1	1362	453	50.39	5.03
TwChi3	mikado.scaffold_10G382.1	1116	371	40.02	6.00
TwChi4	mikado.scaffold_10G599.1	4011	1336	146.91	4.46
TwChi5	mikado.scaffold_11G224.1	2748	915	99.54	4.74
TwChi6	mikado.scaffold_12G339.1	10,833	3610	390.98	4.10
TwChi7	mikado.scaffold_14G114.1	4422	1473	160.22	4.55
TwChi8	mikado.scaffold_17G236.1	2565	854	92.34	4.67
TwChi9	mikado.scaffold_1G646.1	8724	2907	319.50	4.25
TwChi10	mikado.scaffold_21G148.1	6921	2306	249.20	4.32
TwChi11	mikado.scaffold_21G350.2	4473	1490	160.06	4.79
TwChi12	mikado.scaffold_21G351.1	4353	1450	155.53	4.63
TwChi13	mikado.scaffold_26G169.1	2442	813	87.57	4.51
TwChi14	mikado.scaffold_27G208.3	2337	778	83.62	4.40
TwChi15	mikado.scaffold_2G1055.1	2100	699	73.78	4.29
TwChi16	mikado.scaffold_2G1056.1	1905	634	67.60	4.41
TwChi17	mikado.scaffold_2G568.1	2655	884	94.08	4.33
TwChi18	mikado.scaffold_2G719.1	3369	1122	123.54	4.28
TwChi19	mikado.scaffold_30G74.1	4911	1636	179.35	4.49
TwChi20	mikado.scaffold_35G110.1	7548	2515	27.68	4.58
TwChi21	mikado.scaffold_38G186.2	6513	2170	233.58	4.52
TwChi22	mikado.scaffold_3G605.1	6159	2052	222.97	4.32
TwChi23	mikado.scaffold_4G320.1	2514	837	88.54	4.74
TwChi24	mikado.scaffold_5G227.1	3507	1168	125.86	4.37
TwChi25	mikado.scaffold_5G537.1	2586	861	94.28	4.60
TwChi26	mikado.scaffold_5G910.1	2025	674	71.74	4.75
TwChi27	mikado.scaffold_62G1.1	2100	699	73.90	4.14
TwChi28	mikado.scaffold_62G2.1	1941	646	68.94	4.38
TwChi29	mikado.scaffold_6G457.2	5508	1835	199.91	4.26
TwChi30	mikado.scaffold_6G726.1	1836	611	66.72	4.71
TwChi31	mikado.scaffold_7G365.1	2478	825	90.28	4.30
TwChi32	mikado.scaffold_7G450.2	4545	1514	160.16	4.77
TwChi33	mikado.scaffold_23G5.2	2337	778	83.72	4.33
TwChi34	mikado.scaffold_24G266.1	6570	2189	233.94	3.61
TwChi35	mikado.scaffold_2G143.2	1332	443	48.45	6.19
TwChi36	mikado.scaffold_6G219.1	1137	378	40.80	9.19
TwChi37	mikado.scaffold_6G220.1	1557	518	56.98	5.00
TwChi38	mikado.scaffold_48G26.1	657	218	24.34	4.77

**Table 2:** Sequence information of TwChi2 in terms of gene/protein structure, secondary structure, subcellular localization, and activities. CDS, coding sequences.

Name	TwChi2
Molecular weight (KDa)	50.38808
Number of amino acids	453
Length of CDS (bp)	1362
Isoelectric point (pI)	5.03
Instability index	34.43
α-helix (%)	17.44
Extended strand (%)	6.84
β-turn (%)	0
Number of predicted TMHs	0
Conserved domain	Glyco_hydro_19
Subcellular location	Vacuole

## Data Availability

The datasets generated during the current study are available in the NCBI repository, SRR32271522.

## Supplementary Materials

The following supporting information can be downloaded at: https://www.mdpi.com/article/10.3390/md23040144/s1, Table S1: Chitinase sequence of 38 strand *Thalassiosira weissflogii*. Table S2: Analysis and prediction of signal peptides, Transmembrane helices (TMHs) and Subcellular localizations of the TwChi proteins. Table S3: List of the putative motifs of the TwChi proteins. Table S4: The proportion of secondary structure of the TpChi proteins. Table S5: Top 3D models predicted of the TpChi proteins. Table S6: Three diatom-derived chitinases with high homology to TwChi2 obtained from the PLAZA database. Table S7: Sequence information of TwChi2 and three diatom-derived chitinases with high homology to TwChi2 obtained from the PLAZA database. Table S8: List of the putative motifs of the TwChi2 and three diatom-derived chitinases with high homology to TwChi2 obtained from the PLAZA database proteins. Table S9: The exochitinase and endochitinase sequences retrieved in NCBI database. Table S10: The source data of enzyme activity under different substrates and standard curves of different concentrations of *N*-acetylglucosamine. Table S11: The source data of enzyme activity for optimal pH. Table S12: The source data of enzyme activity for optimal temperature. Table S13: The full names of domain abbreviations. Table S14: Keywords used for searching *T. weissflogii* chitinase genes in Tw transcriptome database. Figure S1: Phylogenetic relationships and multiple sequence alignment of the 25 chitinases (Chis) from *Thalassiosira oceanica* (To), *Thalassiosira pseudonana* (Tp), *Cyclotella cryptica* and *Thalassiosira weissflogii* (Tw). Figure S2: Distribution of conserved motifs in the *T. weissflogii* chitinases. Figure S3: Phylogenetic relationships of TwChi2 with 3 endochitinases and 5 exochitinases. Figure S4: Original SDS-PAGE and Western blot of recombinant proteins. Figure S5: UPLC-Q-TOF-MS-MS chromatograms.
