# Supplementary material for: Genome-Wide Mining of Chitinase Diversity in the Marine Diatom Thalassiosira weissflogii and Functional Characterization of a Novel GH19 Enzyme
Source: Mar Drugs. 2025 Mar 26;23(4):144. doi: 10.3390/md23040144 (PMC12028343; doi:10.3390/md23040144)
Supplement: Supplementary file 1 [file marinedrugs-23-00144-s001.zip › Supplementary Materials/Figures S1-S5.pptx]

## Slide 1
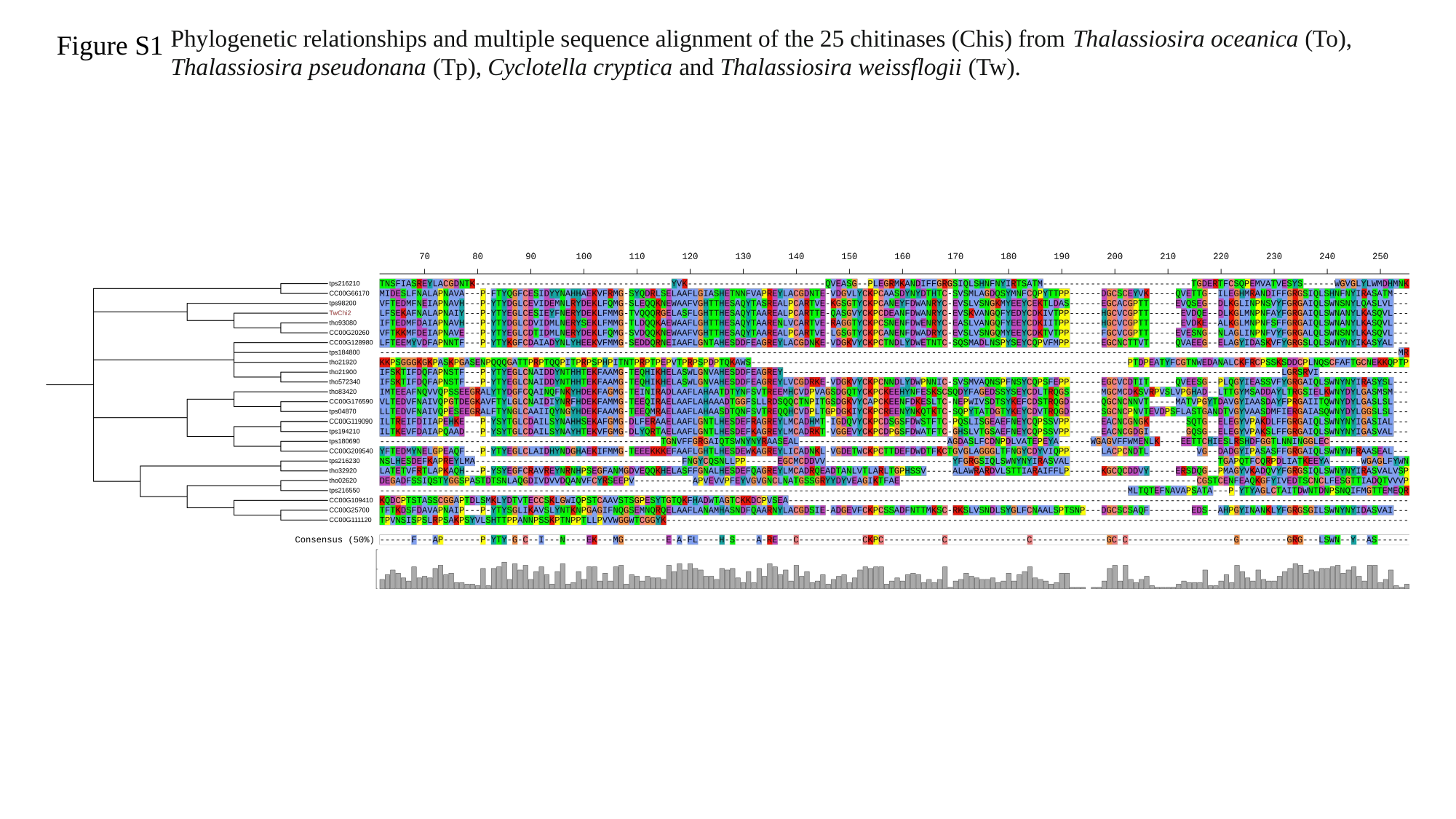

Phylogenetic relationships and multiple sequence alignment of the 25 chitinases (Chis) from Thalassiosira oceanica (To), Thalassiosira pseudonana (Tp), Cyclotella cryptica and Thalassiosira weissflogii (Tw).
Figure S1

## Slide 2
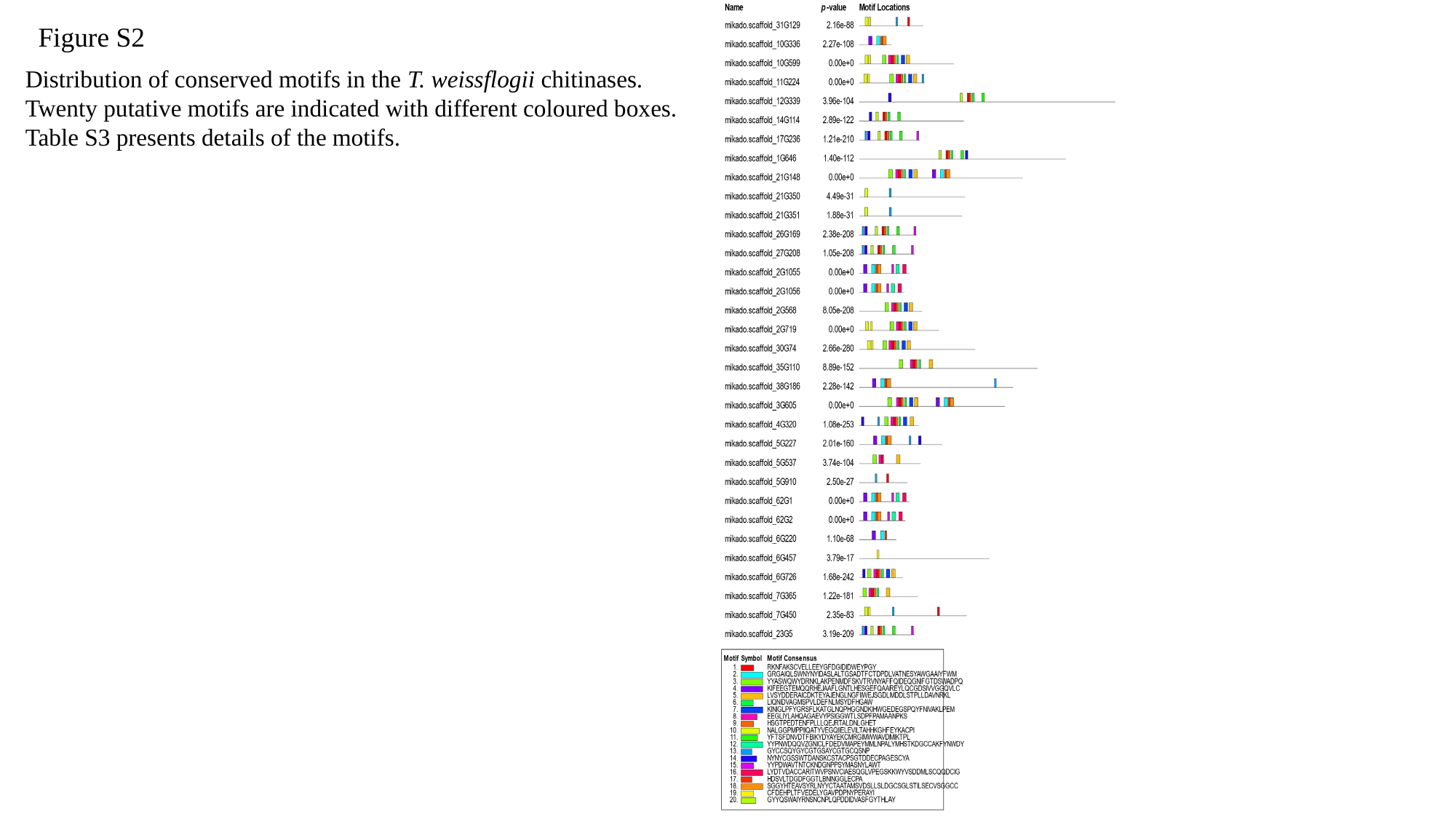

Figure S2
Distribution of conserved motifs in the T. weissflogii chitinases. Twenty putative motifs are indicated with different coloured boxes. Table S3 presents details of the motifs.

## Slide 3
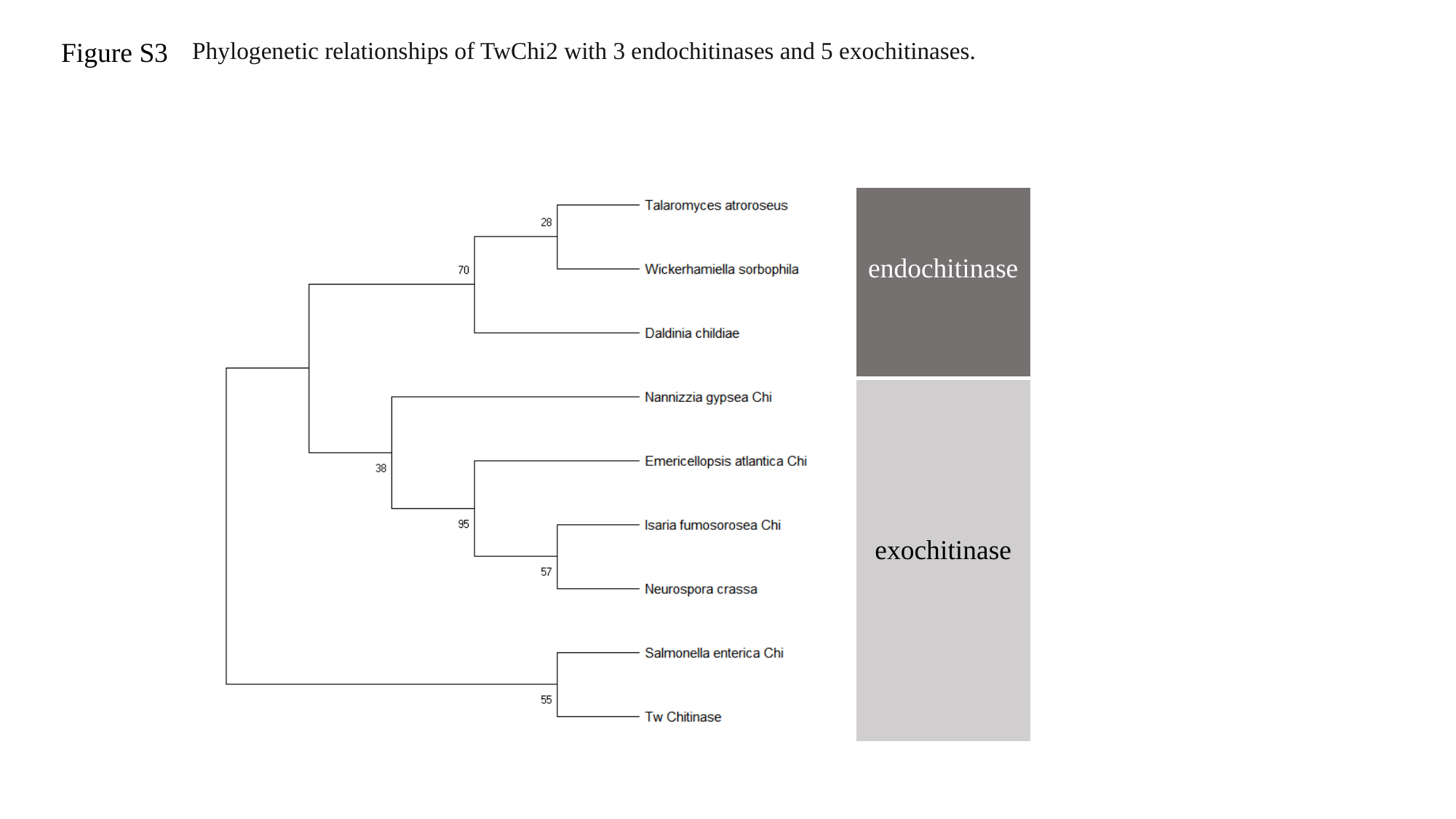

Figure S3
Phylogenetic relationships of TwChi2 with 3 endochitinases and 5 exochitinases.
| endochitinase |
| --- |
| exochitinase |

## Slide 4
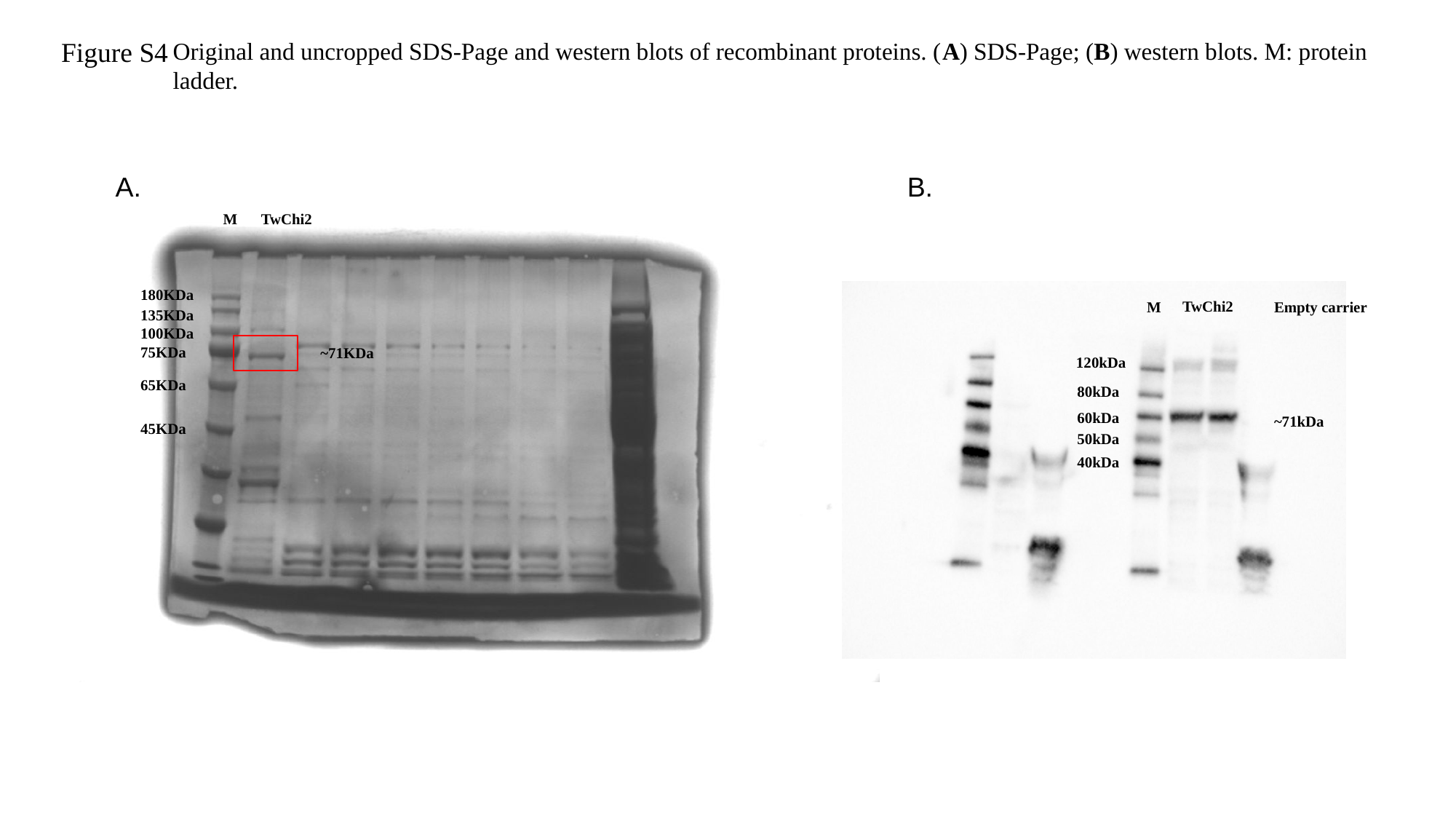

Figure S4
Original and uncropped SDS-Page and western blots of recombinant proteins. (A) SDS-Page; (B) western blots. M: protein ladder.
B.
A.
M
TwChi2
180KDa
TwChi2
M
Empty carrier
135KDa
100KDa
75KDa
~71KDa
120kDa
65KDa
80kDa
60kDa
~71kDa
45KDa
50kDa
40kDa

## Slide 5
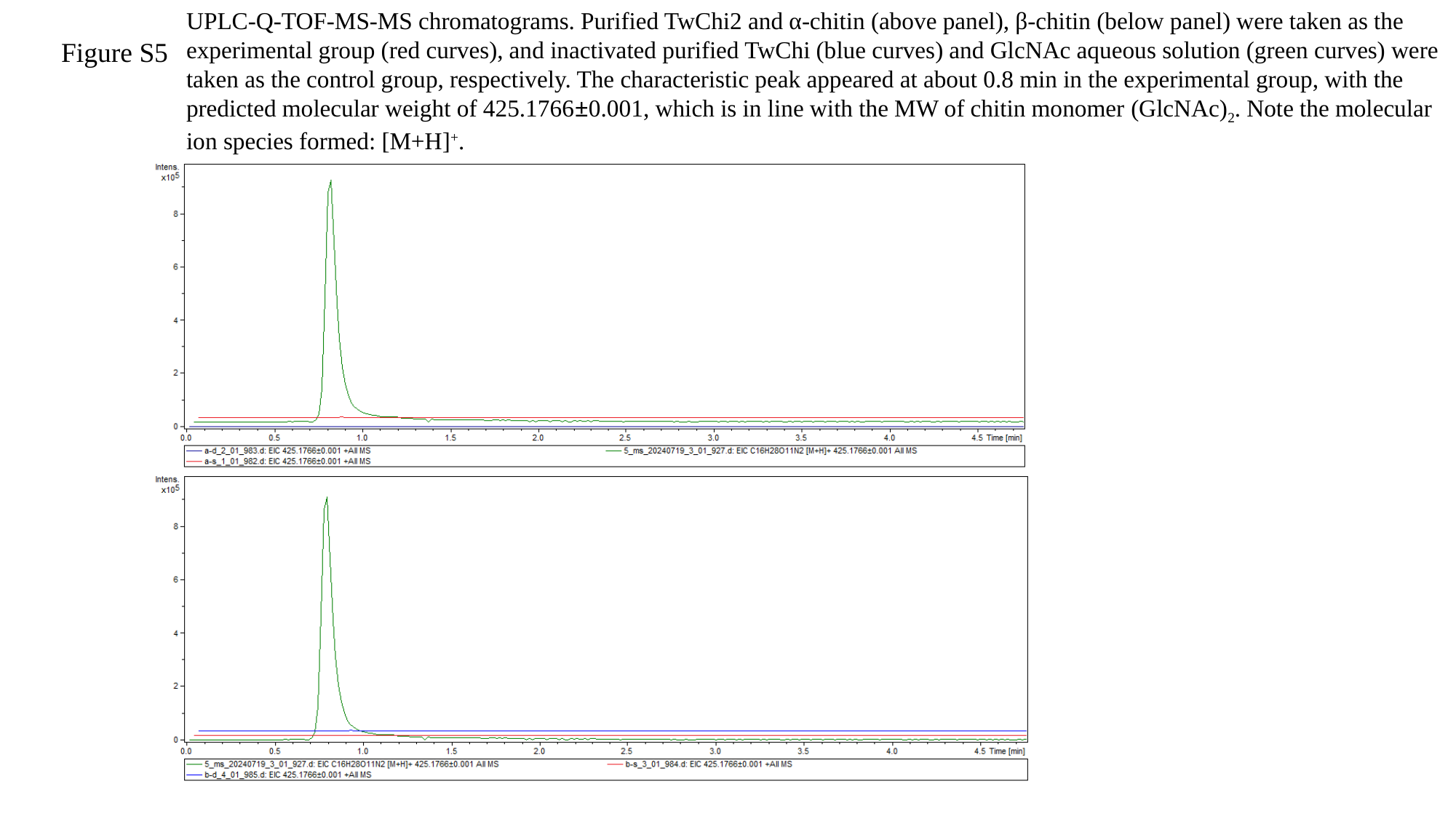

UPLC-Q-TOF-MS-MS chromatograms. Purified TwChi2 and α-chitin (above panel), β-chitin (below panel) were taken as the experimental group (red curves), and inactivated purified TwChi (blue curves) and GlcNAc aqueous solution (green curves) were taken as the control group, respectively. The characteristic peak appeared at about 0.8 min in the experimental group, with the predicted molecular weight of 425.1766±0.001, which is in line with the MW of chitin monomer (GlcNAc)2. Note the molecular ion species formed: [M+H]+.
Figure S5
